# Comparative evaluation of osteogenic differentiation potential of stem cells derived from dental pulp and exfoliated deciduous teeth cultured over granular hydroxyapatite based scaffold

**DOI:** 10.1186/s12903-021-01621-0

**Published:** 2021-05-15

**Authors:** Manal Nabil Hagar, Farinawati Yazid, Nur Atmaliya Luchman, Shahrul Hisham Zainal Ariffin, Rohaya Megat Abdul Wahab

**Affiliations:** 1 Department of Family Oral Health, Faculty of Dentistry, Universiti Kebangsaan Malaysia, Jalan Raja Muda Abdul Aziz, 50300 Kuala Lumpur, Malaysia; 2 School of Bioscience and Biotechnology, Faculty of Science and Technology, Universiti Kebangsaan Malaysia, 43600 Bangi, Selangor Malaysia

**Keywords:** Granular hydroxyapatite scaffold, Stem cells from exfoliated deciduous teeth (SHED), Dental pulp stem cells (DPSC), MC3T3-E1 cells, Osteogenesis

## Abstract

**Background:**

Mesenchymal stem cells isolated from the dental pulp of primary and permanent teeth can be differentiated into different cell types including osteoblasts. This study was conducted to compare the morphology and osteogenic potential of stem cells from exfoliated deciduous teeth (SHED) and dental pulp stem cells (DPSC) in granular hydroxyapatite scaffold (gHA). Preosteoblast cells (MC3T3-E1) were used as a control group.

**Methodology:**

The expression of stemness markers for DPSC and SHED was evaluated using reverse transcriptase-polymerase chain reaction (RT-PCR). Alkaline phosphatase assay was used to compare the osteoblastic differentiation of these cells (2D culture). Then, cells were seeded on the scaffold and incubated for 21 days. Morphology assessment using field emission scanning electron microscopy (FESEM) was done while osteogenic differentiation was detected using ALP assay (3D culture).

**Results:**

The morphology of cells was mononucleated, fibroblast-like shaped cells with extended cytoplasmic projection. In RT-PCR study, DPSC and SHED expressed GAPDH, CD73, CD105, and CD146 while negatively expressed CD11b, CD34 and CD45. FESEM results showed that by day 21, dental stem cells have a round like morphology which is the morphology of osteoblast as compared to day 7. The osteogenic potential using ALP assay was significantly increased (*p* < 0.01) in SHED as compared to DPSC and MC3T3-E1 in 2D and 3D cultures.

**Conclusion:**

gHA scaffold is an optimal scaffold as it induced osteogenesis in vitro. Besides, SHED had the highest osteogenic potential making them a preferred candidate for tissue engineering in comparison with DPSC.

## Introduction

Bone defects due to congenital abnormalities, incidents such as fractures, diseases (osteoporosis and osteosarcoma) or trauma are problems that many people have suffered. Alveolar bone graft is a significant part of the treatment of congenital fissure which is caused by different hereditary and environmental factors that anticipate the palatal shelves to fuse appropriately around the fifth and the sixth week of gestation [1]. According to many studies, the autologous bone graft is the best treatment for repairing alveolar cleft, however, it is limited by donor site morbidity and insufficient supply of autologous bone. The failure rate of autologous bone graft is around 15% or more and it has unfavorable results [2]. As an alternative method for autogenous and allogeneic grafts, a tissue engineering approach is used to improve the clinical outcome of the alveolar cleft patient by augmenting the alveolar bone or by filling the bone defect.

Stem cells and scaffolds have an essential role in tissue engineering as the stem cell acts as the seed and the scaffold acts as an extracellular matrix for bone regeneration. The need for more accessible mesenchymal stem cells (MSCs) than those found in bone marrow has increased interest in dental tissues that are a rich source of stem cells. Stem cells from exfoliated deciduous teeth (SHED) and dental pulp stem cells (DPSC) were used in this study due to their ease of extraction and these cells exhibit a high osteogenic potency [3]. Asutay et al. [4] and Khanna Jain et al. [5] have reported that DPSC have significant osteogenic differentiation ability for bone regeneration. They have a great role in tissue engineering because of their ability to be cryopreserved, attach with many scaffolds, low morbidity after collection and anti-inflammatory abilities [6]. DPSC is an alternative source of bone marrow stem cells (BMSC) for bone regeneration since it shows a higher proliferation and osteogenic potential as compared with BMSC [7].

For effective tissue engineering, several criterias should be available in the scaffold. An ideal scaffold must have the ability to interact with the stem cell and should be porous to accommodate cell proliferation and differentiation. The scaffold should be non-immunogenic and provide a 3D environment that can enhance the proliferation of stem cells. It should also promote vascular growth and has the ability to undergo biodegradation. The scaffold or three-dimensional (3D) construct allows the cells to proliferate, maintain their differentiated function and provide support for new bone formation [8]. Typically, the scaffolds have three types including ceramics, natural polymers and synthetic polymers for tissue engineering [9]. Composite scaffolds consist of various ceramic, natural and synthetic polymers. The drawbacks of natural scaffold include disease transmission and sterilization while the disadvantages with the synthetic polymers are the chronic inflammatory response, complex architecture and toxic degradation [9]. Ceramic scaffolds such as hydroxyapatite (HA) and tri-calcium phosphate (TCP) are typically characterized by many features such as bioactivity, high mechanical stiffness (Young’s modulus), very low elasticity, osteoconductivity, non-toxic and similar with natural bone in the chemical structures. It also has more potential for stem cell-based bone engineering by providing the highest cells adhesion and proliferation [6, 10].

Studies on granular hydroxyapatite scaffold had demonstrated to be exceedingly osteoconductive and suitable in the development of mature bone over the deformity [11]. Granular hydroxyapatite scaffold is recommended as a filler of bone defects, a bridge or spacer of regions with bone loss and for augmentation, correction and rectification of malpositioned bone. However, the behavior of DPSC and SHED in a granular hydroxyapatite scaffold have not been studied. This paper demonstrates the osteogenic differentiation of MC3T3-E1, SHED and DPSC that are seeded in granular hydroxyapatite scaffold.

## Material and method

The study was performed using primary and permanent teeth from children and adults. Ethical approval was obtained from the Ethical Committee University Kebangsaan Malaysia (No: UKM PP/111/8/JEP-2017-549). Progenitor cells of the MC3T3-E1 subclone C14 (MC3T3-E1/C14) preosteoblast cell line (ATCC No: CRL-2596TM) were used in this study as a control side. Human dental pulp was extracted from exfoliated deciduous teeth and permanent teeth. The tooth extraction was performed in the Faculty of Dentistry, University Kebangsaan Malaysia (UKM) and Hospital Canselor Tuanku Muhriz (HCTM).

### Isolation and culture of DPSC and SHED

The dental pulp tissue from extracted teeth was cut into small pieces using a scalpel blade before digesting it in a solution of 3 mg/ml collagenase type 1 (Sigma-Aldrich, Inc) for 30 min until a cotton appearance was obtained. Followed with the addition of 1 ml of the culture medium to inhibit the enzyme activity. The culture medium of DPSC and SHED was shown in Table 1. After that, The cells were centrifuged at 1200 g for 6 min at 25 °C. The pellet later was mixed with 1 ml of culture medium and transferred to T75 cm^2^ culture flasks. Finally, the cells were incubated at 37 °C with a humidity of 95% and 5% CO_2_ [12].

**Table 1:** The components and the amounts of the prepared medium for MC3T3-E1, DPSC, and SHED (standard medium)

Material type	Description
Media preparation for MC3T3-E1 cells	Alpha modified Eagle’s medium (α-MEM) (Invitrogen, USA) supplemented with 1 mM sodium pyruvate, 10% (v/v) fetal bovine serum (FBS) (Biowest, South America) and 1% (v/v) penicillin/streptomycin (Invitrogen, USA) were used as a complete media
Media preparation for DPSC and SHED	Dulbecco’s modified Eagle’s medium (DMEM) (Invitrogen, USA) supplemented with 1% Glutamax, 10% (v/v) fetal bovine serum (FBS) (Biowest, South America) and 1% (v/v) penicillin/streptomycin (Invitrogen, USA) were used as a complete media

### Characterization of MC3T3-E1, DPSCs, SHED in 2-dimensional flask.

#### Morphological analysis

The morphology of MC3T3-E1, DPSC and SHED was observed under a light microscope (Olympus CK30, USA) using cell B software to record the morphology of the cells throughout the culture.

#### Reverse transcription polymerase chain reaction (RT-PCR) analysis for detecting the stemness markers of DPSC and SHED

Total RNA was extracted from two flasks of T25 (80% confluent). One flask contained DPSC and the other one contained SHED. Total RNA was separated from the cells using 1 ml 100% Trizol (Sigma, USA) for 5 min at room temperature to lyse the cells. Then, 200 µl of chloroform added and the sample was shaken vigorously for 15 s. The sample was left for 10 min at room temperature and centrifuged at 1200 g for 15 min at 4 °C. After centrifugation, three layers can be observed that are protein (organic phase), DNA (middle layer), and RNA (colorless phase) were obtained. The aqueous stage was evacuated and moved to a DEPC-treated cylinder. About 500 µl of 100% (v/v) isopropanol was added to the aqueous stage. Then, it was left for 10 min at room temperature before it was centrifuged at 1200 g at 4 °C for 10 min. The supernatant was evacuated and afterward, 1 ml of 75% (v/v) cold ethanol was added to the pellet for better RNA quality. The mixture was vortexed and centrifuged at 7500 g at 4 °C for 5 min. The pellet was left to dry and then it was resuspended in 20–50 µl of nuclease-free water. It was incubated in a water bath 55 °C and stored at − 80 °C. The concentration of RNA was distinguished by biophotometer (Eppendorf, Germany) [13].

For every RT-PCR, two hundred nanograms of RNA was used for synthesis cDNA template. One µg of DNA obtained from RNA extraction was used as a template. The RT-PCR (kit AccessQuick RT-PCR system, Promega, USA) contains 5 × green or colorless GoTaq® Flexi Buffer reaction (4 µl), magnesium chloride (MgCl_2_), forward and reverse primers (0.8 µl), dNTP mix (0.4 µl) and DNA polymerase (1 µl) and Taq* (0.25 µl). RT- PCR reaction for DPSC and SHED was conducted using a specific pair of primers (i.e., GAPDH, CD73, CD105, CD146, CD34, CD45, and CD11b) as shown in (Table 2). One cycle (45 min at 45 °C) was used for reverse transcription. This is followed by one cycle (2 min at 94 °C). Then, 35 cycles of denaturation, annealing and extension (94 °C for 30 s, 55 °C for 1 min, and 68 °C for 2 min; respectively) were done for secondary cDNA synthesis and PCR amplification. A final extension cycle through at 68 °C for 7 min. Agarose gel was prepared from 0.34 g of agarose powder (Promega, USA) and 20 µl of Tris–acetate buffer. It is used for separating the amplified products. The gels were stained with ethidium bromide and analyzed using UV Alpha Imaging System (Alpha Innotech, Cell Bioscience).

**Table 2:** The sequence and length of the primers (RT-PCR)

Name	Forward primer	Reverse primer	Length (bp)
GAPDH	TCCATGACAACTTTGGTATCG	TGTAGCCAAATTCGTTGTCA	471
CD73	TGATAATGGTGTGGAAGGACTG	TGCTTGGATCTTCAGGAATGC	599
CD105	CGGTGAAGGTGGAACTGA	TTCCGCTGTGGTGATGAG	610
CD146	CCACCACACTTCAGCATCA	CACAAGACAGATTCAACACCAT	523
CD34	CAAGTTAGTAGCCAACGAG	GCTTCAAGGTTGTCTCTGGAG	589
CD45	AACCGAATCTGACATCATCACC	AGCAGGCACAAGAAGGTAGG	504
CD11b	TCTTGATTGATGGCTCTGGTAG	GGCTTGGATGCGATGGTATT	463

### Seeding of SHED and DPSC in a granular hydroxyapatite scaffold

Hydroxyapatite scaffold granules (Granumas, Malaysia) were set as a monolayer at the base of a well in 24-well plates and presoaked in complete medium overnight to minimize ion release from the particles in 24 well plates. The scaffold was dried in a CO_2_ incubator for 24 h at 37 °C. Each layer of granular hydroxyapatite covering one of 24 wells was seeded with 5 × 10^4^cells/cm^3^ in 200 µl of culture medium. The plates were incubated for a day at 37 °C permitting the attachment of the cells. On a subsequent day, half samples were cultured in 500 µl of standard medium (complete medium) while the other half were cultured in differentiating medium (standard medium in addition to 10 nm β-glycerophosphate and 50 µg/ml ascorbic acid). Finally, the cell culture plate was set in an incubator with a humidified air of 95% air, 5% CO_2_ at 37 °C for 10 days. The medium was changed every three days.

#### Osteoblast differentiation

The differentiating factor was added to the complete growth medium during cell plating in 24 well plates for the differentiation of dental pulp stem cell. Approximately 5 × 10^4^ cells were prepared in each of the wells for osteoblastic differentiation analysis. Then, 50 µg/ml ascorbic acid (Sigma, USA) and 10 mM β-glycerol phosphate (w/v) (Sigma, USA) were added. The medium was changed every three days. The complete medium without induction factor was used as a negative control [13].

#### Sample preparation of MC3T3-E1, DPSC and SHED after seeding in gHA scaffold for scanning electron microscope

The cells were seeded in a complete medium (standard medium) and in an osteogenic medium (differentiating medium) for 7 and 21 days. The seeded scaffolds were washed and fixed in 2.5% glutaraldehyde for one night. At that point, the samples were washed multiple times for 10 min with PBS and dehydrated in a graded concentration of alcohol 35%, 50%, 70%, 80% for 10 min. Then, it was dehydrated three times in 100% ethanol. After drying out, the sample was dried with the Critical Point Dryer machine. The drying procedure included a solution of acetone and carbon dioxide gas. After that, the samples were sputtered in a sputter coater (SCD 005, BAL-TEC). Finally, the scaffold with or without the cells was seen under a scanning electron microscope [10].

#### Alkaline phosphatase assay of MC3T3-E1, DPSC and SHED

For harvesting the cells for alkaline phosphatase assay (ALP), the old medium was removed. Then, the attached cells were washed tenderly with a cold titrate buffered saline (TBS). The TBS was removed. After that 30 µl of lysate buffer was added to each well. Lysate buffer was prepared from 40 µl Trion X100 (Sigma, USA) and 49 ml of TBS. For the samples without a scaffold, the bottom of each well plate was scratched gently by pipettes and it was transferred into a new 1.5 ml microtube. For the sample with scaffold, however, the well plates were sealed above the ice with parafilm and shaken in a shaking incubator for 30 min. After that, each sample was transferred gently by pipettes into a different 1.5 ml microtube.

For assessment of alkaline phosphate activity, the tubes were centrifuged two times at 1600 g, 4° for 10 min. About 10 µl of cells was set in every 96 well plates. Then, 40 µl of MgSO_4_ (w/v) (Sigma, USA), 10 µl of p-nitrophenyl phosphate (w/v) (Sigma, USA) and 40 µl of bicarbonate-carbonate buffer (NaNo_3_-Na_2_Co_3_) (pH 10.0) (w/v) (R&M, U.K) were added to each 96 well plate as shown in Table 3. Then, the cells were incubated for 30 min at 37 °C. After incubation, 100 µl of NaOH was added to each sample to stop the enzymatic reaction. The optical density reading was taken at wavelength 405 nm with a spectrophotometer [13].

**Table 3:** The concentration of alkaline phosphatase analysis components

Material type	Concentration
5 mM MgSO_4_	12.3 mg of MgSO_4_ + 10 ml ddi (from this mixture, 40 µl for each well)
Bicarbonate-carbonate buffer Na_2_CO_3_ + NaHCO_3_ (PH 10)	Na_2_CO_3_ = 317.97 mg + 15 ml ddi NaHCO_3_ = 105.01 mg + 15 ml ddi 10.5 ml of Na_2_CO_3_ + 4.5 ml of NaHCO_3_ (from this mixture, 40 µl for each well plate)
P-nitrophenyl phosphate (PNP-P)	2.782 mg + 2 ml ddi (from this mixture, 10 µl for each well plate)
Sodium hydroxide (NaOH)	1.6 mg of NaOH + 20 ml ddi ( from this mixture, 100 µl for each well plate)

The analysis was performed on cells which were cultured in differentiated mediums on day 0, 7, 14, and 21 for 3D culture [10, 14] while it was performed on day 0, 3, 6, 9, 12, 15, 18 and 21 for 2D culture. The activity of ALP enzyme was expressed in a unit (U). One unit is equivalent to 1 μmol p- nitrophenol which is released per minute at 37 °C. The specific activity of ALP (U/mg) is obtained by dividing the enzyme activity units with the amount of protein (mg). The amount of protein was determined by using the standard curve of the Bradford method. Cellular ALP activity was determined by plotting a graph of specific activity against days of analysis [15].

### Statistical analysis

Experiments were carried out in triplicate for cells derived from five donor samples. The data are exhibited as mean ± standard deviation (SD) for every single test. Statistically, a comparison between the data of DPSC and SHED with the control group (MC3T3-E1) at different days was done by repeated measure two-way ANOVA (Bonferroni post hoc). All the statistical analysis was performed at the significance level *p* < 0.01. One way (ANOVA) with Bonferroni post hoc test for multiple comparisons was used to compare the differences in the days for ALP activity [5].

## Results

### Morphological analysis of MC3T3-E1, DPSC and SHED

**Fig. 1:**
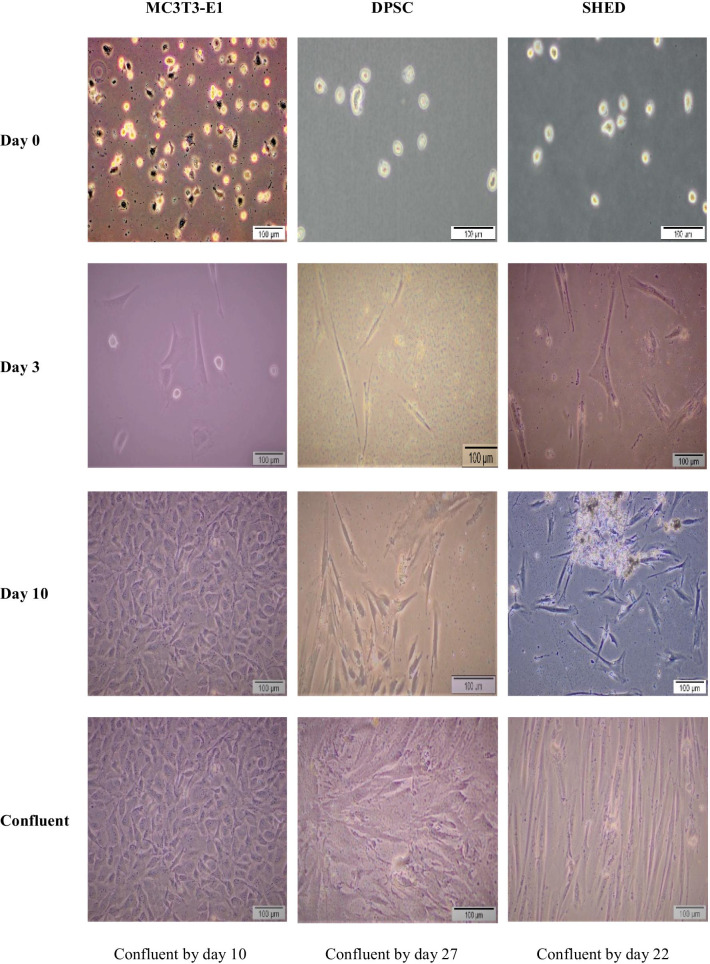
The morphology of MC3T3-E1, DPSC, and SHED at day 0,3,10 using a light microscope (Magnification 100×)

Cells extracted from dental pulp tissue using digestion enzymes were initially observed using light microscope in a sphere form. After 48 h, the spherical cell's morphology changes into a fibroblastic form (Fig. 1). At day 10, DPSC and SHED started to become semi-confluent at about 50% confluency while MC3T3-E1 began to become confluent at about 90% confluency. SHED become confluent by day 22 while DPSC by day 27.

### RT-PCR analysis for DPSC and SHED

Stem cell markers were used to recognize and identify the stem cells which were isolated from the human pulp tissue. CD34, CD45 and CD11b were used as a negative control because those markers are specific markers for hematopoietic stem cells, leukocytes, and macrophages respectively [16–20].

**Fig. 2:**
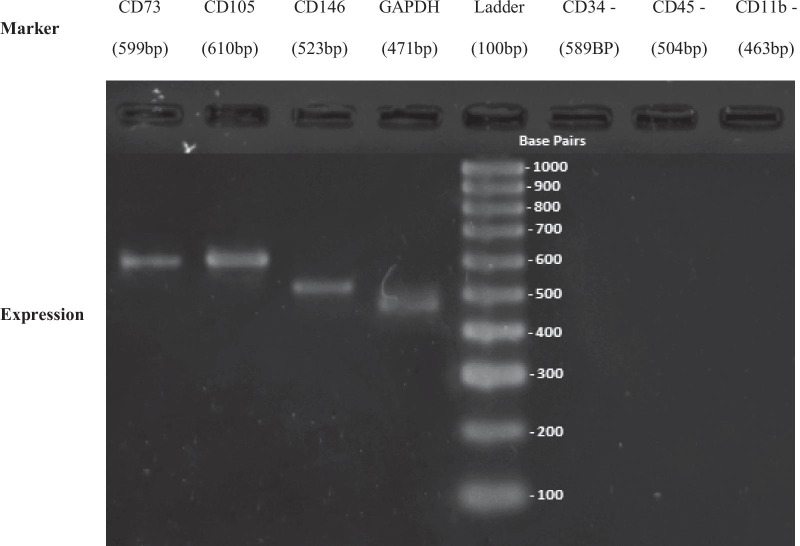
Positive MSC markers and negative HSC markers of DPSC using RT-PCR

**Fig. 3:**
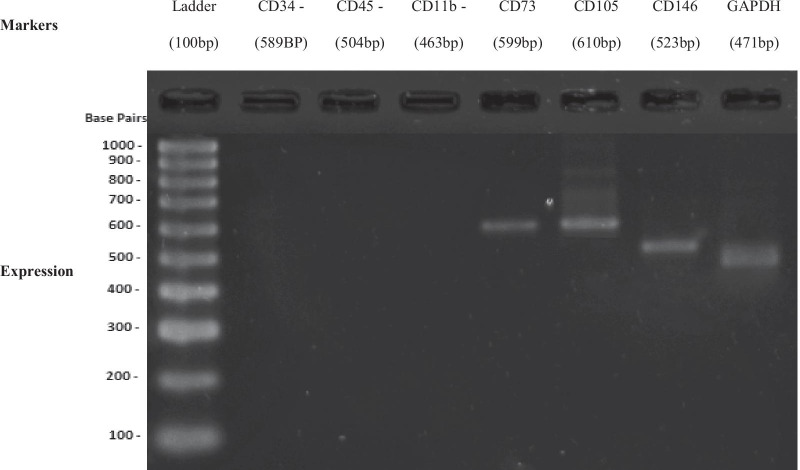
Positive MSC markers and negative HSC markers of SHED using RT-PCR

RT-PCR analysis in our results proved that DPSC and SHED expressed mesenchymal stem cell markers like CD73, CD105 and CD146 while these cells did not express CD34, CD45, and CD11b. This was indicated that these cells are of mesenchymal origin (Figs. 2, 3).

### Seeding of SHED and DPSC in a granular hydroxyapatite scaffold

The osteogenic differentiation of DPSC and SHED in a granular hydroxyapatite scaffold was evaluated by SEM and ALP assay.

#### Morphology of MC3T3-E1, DPSC and SHED in gHA scaffold using scanning electron microscope

**Fig. 4:**
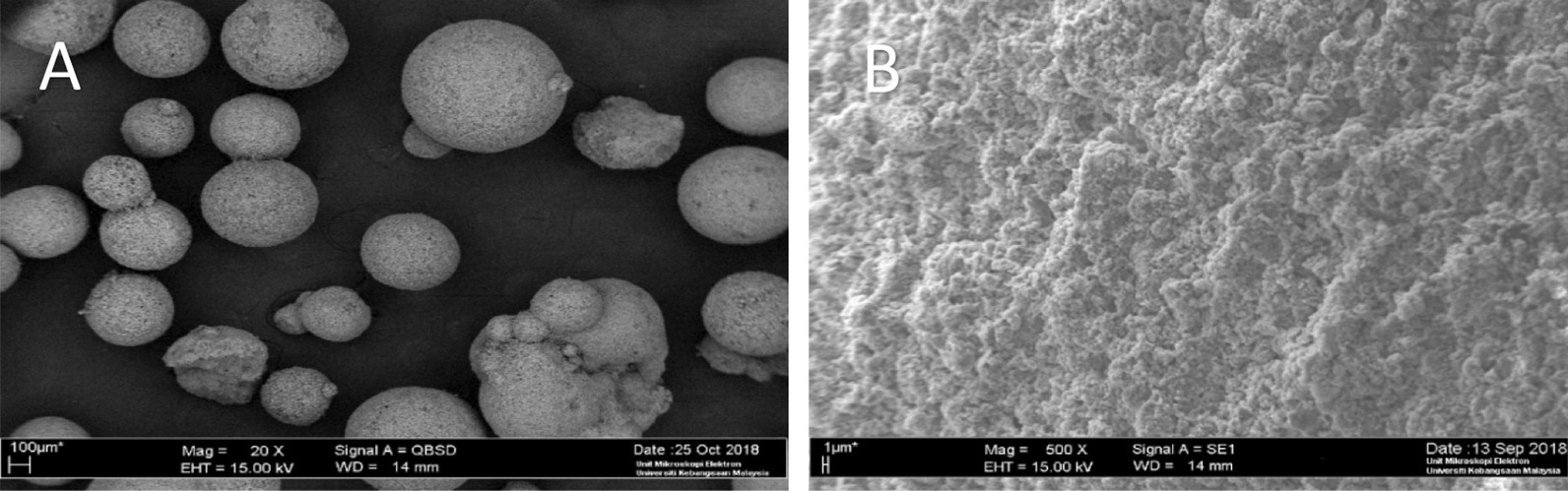
SEM micrograph of a cross-sectional view of a granular hydroxyapatite scaffold at different magnification 20× and 500×

**Fig. 5:**
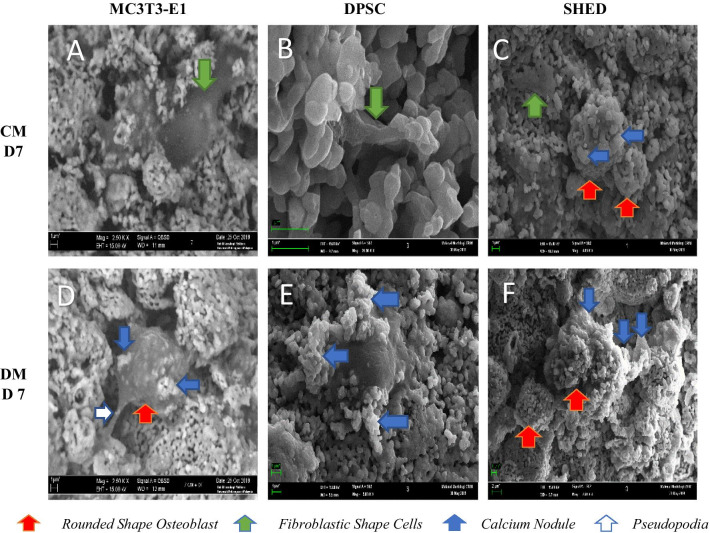
SEM micrograph of a granular hydroxyapatite scaffold seeded with MC3T3-E1, SHED and DPSC at day 7. (**a**, **b**, **c**) in complete medium only while (**d**, **e**, **f**) in a differentiate medium at 2500× magnification and at scale bar 1 µm. The blue arrows showed that more calcium nodules were observed in the differentiate medium. The green arrows showed the fibroblastic shape cells in complete medium while the red arrows showed the rounded shape osteoblasts in the differentiated medium. CM = complete medium, DF = differentiating medium

**Fig. 6:**
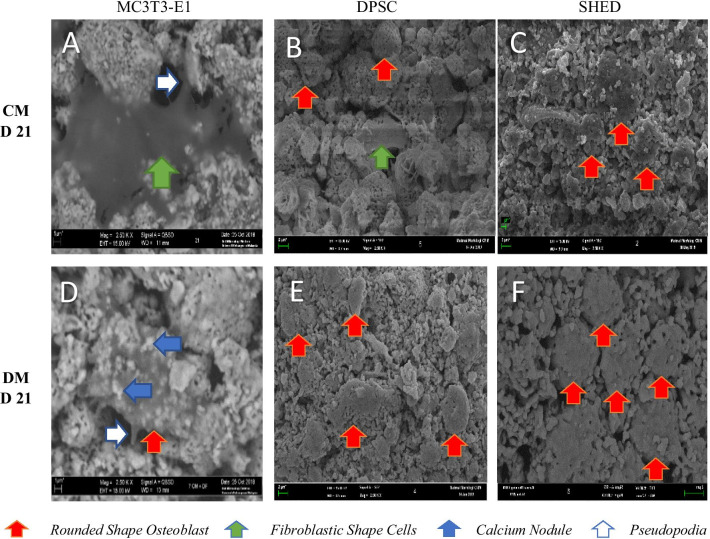
SEM micrograph of a granular hydroxyapatite scaffold seeded with MC3T3-E1, DPSC and SHED cells on day 21. (**a**, **b**, **c**) in complete medium only while (**d**, **e**, **f**) in a differentiated medium at 2500× magnification. The blue arrows showed that more calcium nodule (white patches) were observed in the differentiated medium. The green arrows showed the fibroblastic shape cells while the red arrows showed the rounded shape osteoblasts. White arrows showed the pseudopodia. CM = complete medium, DF = differentiating medium

The unloaded scaffold had a bumpy surface in Fig. 4 while fibrous-like organic structures were observed in the loaded scaffold. The shape of the cells was polygonal with elongated cytoplasmic extensions which indicated the adhesion of cells. In a complete medium, the cells showed a fibroblastic like morphology while using the differentiating medium; the osteoblastic-like morphology, round-like shape, was observed on cell-attached on the granular hydroxyapatite scaffold (Figs. 5, 6). Cells’ attachment to the scaffold granules was evident by Day 7. As incubation day increased, there were cell-to-cell and cell-to-adjacent-granule attachments through pseudopodia (white arrow).

The three types of cells were observed to continuously proliferate following days of incubation. At higher magnification, single flatten cell with finger-like projection can be observed on day 7 for MC3T3-E1 and DPSC using complete medium while for SHED, rounded shaped osteoblast was observed on day 7 with precipitation of mineralized nodule adjacent to DPSC and SHED (Fig. 5a–c). SHED had the highest abundance of mineralized nodules even in the standard medium on day 7. By day 21 more irregular shaped osteoblasts were observed in DPSC while for SHED homogeneous rounded like osteoblasts are observed in (Fig. 6d–f). The formation of collagen fibers can be appreciated in the tested group as compared to the control group.

#### ALP analyses of cells seeded in granular hydroxyapatite scaffold

**Fig. 7:**
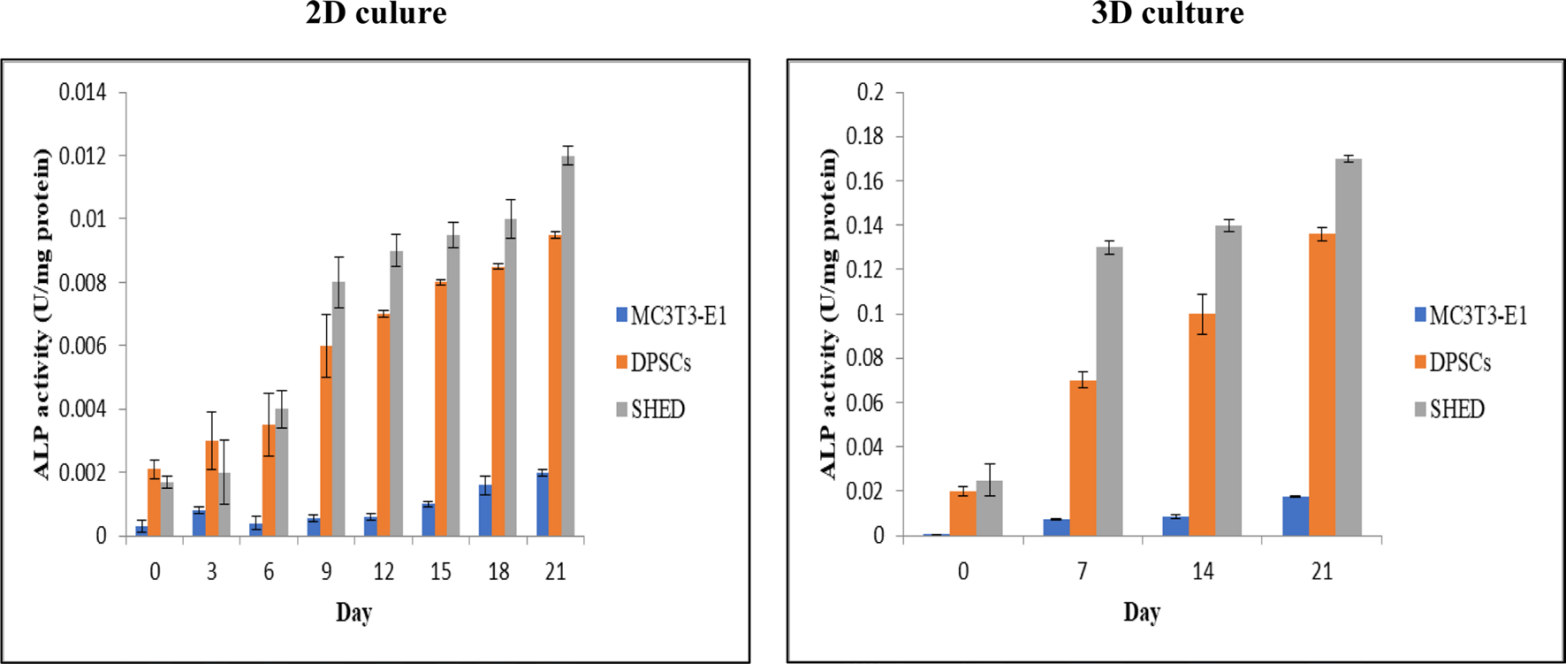
The specific activity of ALP enzyme activity of MC3T3-E1, DPSC, and SHED using cell differentiation medium in 2D and 3D culture

The results in 2D culture after 21 days of culturing DPSC and SHED in differentiating medium, it was shown that the cells were differentiated into osteoblast. The results were assessed on day 0, 3, 6, 9, 12, 15, 18 and day 21 (Fig. 7, Tables 4, 5).There was a significant increase of ALP activity of SHED and DPSC as compared to the control (MC3T3-E1) *p* < 0.01, n = 5. A repeated measure two-way ANOVA (Bonferonni Post Hoc) to compare the ALP activity of MC3T3-E1, DPSCs and SHED without scaffold was done. Results of the ANOVA showed a significant difference in ALP activity between the three types of cells (MC3T3-E1, DPSC, SHED); F (1.14, 18.2) = 213, *p* < 0.01. Bonferonni Post Hoc test revealed that the ALP activity of the SHED group (0.007 ± 0.059) is significantly higher than MC3T3-E1 (0.001 ± 0.007) and also higher than DPSC (0.006 ± 0.045) as shown in (Tables 4, 5).

**Table 4:** Comparison of alkaline phosphatase activity of MC3T3-E1, DPSC and SHED using two-way ANOVA in 2D culture

Type of cell	Comparing with	*p* value	Mean difference ± SD
MC3T3-E1	DPCS	0.02	0.0009 ± 0.0005
	SHED	0.000*	
DPSC	MC3T3-E1	0.02	0.006 ± 0.003
	SHED	0.005*	
SHED	MC3T3-E1	0.000*	0.007 ± 0.004
	DPSC	0.005*	

Values expressed as mean differences ± SD. n = 5, *p < 0.01

**Table 5:** Difference in the average of alkaline phosphase activity between days for all groups in 2D culture. n = 5, *p < 0.01

Observation interval	The average of ALP in differentiating medium		
	Mean difference ± Std	Observation interval with	*p* value
Day 0	0.005 ± 0.0001	Day 3	0.241
		Day 6	0.006
		Day 9	0.000*
		Day 12	0.000*
		Day 15	0.000*
		Day 18	0.000*
		Day 21	0.000*
Day 3	0.002 ± 0.0001	Day 0	0.241
		Day 6	1.00
		Day 9	0.000*
		Day 12	0.000*
		Day 15	0.000*
		Day 18	0.000*
		Day 21	0.000*
Day 6	0.003 ± 0.0004	Day 0	0.006*
		Day 3	1.000
		Day 9	0.002*
		Day 12	0.000*
		Day 15	0.000*
		Day 18	0.000*
		Day 21	0.000*
Day 9	0.005 ± 0.0007	Day 0	0.000*
		Day 3	0.000*
		Day 6	0.002*
		Day 12	1.000
		Day 15	0.137
		Day 18	0.003*
		Day 21	0.000*
Day 12	0.0056 ± 0.0003	Day 0	0.000*
		Day 3	0.000*
		Day 6	0.000*
		Day 9	1.000
		Day 15	1.000
		Day 18	0.128
		Day 21	0.001
Day 15	0.0061 ± 0.0001	Day 0	0.000*
		Day 3	0.000*
		Day 6	0.000*
		Day 9	0.137
		Day 12	1.000
		Day 18	1.000
		Day 21	0.027
Day 18	0.007 ± 0.003	Day 0	0.000*
		Day 3	0.000*
		Day 6	0.000*
		Day 9	0.003*
		Day 12	0.128
		Day 15	1.000
		Day 21	1.000
Day 21	0.008 ± 0.0003	Day 0	0.000*
		Day 3	0.000*
		Day 6	0.000*
		Day 9	0.000*
		Day 12	0.001*
		Day 15	0.027
		Day 18	1.000

In this study, the results also revealed that the granular hydroxyapatite scaffold promoted the differentiation of MC3T3-E1, DPSC, and SHED. The results were also assessed up to 21 days (Fig. 7, Tables 6, 7). The ALP activity of MC3T3-E1, DPSC and SHED in the differentiating medium was increased by day 14 and day 21. A repeated measure two-way ANOVA (Bonferonni Post Hoc) within ALP activity of MC3T3-E1, DPSC and SHED was done (Table 6). Results of ANOVA test revealed that ALP activity of SHED group (0.118 ± 0.059) is significantly higher than MC3T3-E1 (0.006 ± 0.007) and also higher than DPSCs (0.08 ± 0.045) F (1.173, 12.899) = 46.55, *p* < 0.01. This proved that there is a significant difference in ALP activity, and it proved that the SHED had the highest osteogenic potential making them a better candidate for tissue engineering.

**Table 6:** Comparison of alkaline phosphatase activity of MC3T3-E1, DPSC and SHED using two-way ANOVA in 3D culture

Type of cell	Comparing with	*p* value	Mean difference ± SD
MC3T3-E1	DPSC	0.01	0.006 ± 0.007
	SHED	0.000*	
DPSC	MC3T3-E1	0.01	0.08 ± 0.045
	SHED	0.001*	
SHED	MC3T3-E1	0.000*	0.118 ± 0.059
	DPSC	0.001*	

Values expressed as mean differences ± SD. n = 5, *p < 0.01

**Table 7:** Difference in the average of alkaline phosphase activity between days for all groups in 3D culture

Observation interval	Comparing with	Differentiating medium	
		Mean difference ± SD	*p* value
Day 0	Day 7	0.0145 ± 0.0012	0.001*
Day 7	Day 14	0.0686 ± 0.0001	0.03
Day 14	Day 21	0.0849 ± 0.005	0.001*
Day 21	Day 0	0.108 ± 0.001	0.000*

n = 5, *p < 0.01

A one-way ANOVA between the mean of ALP activity of the three groups of cells and the differences in the day was carried out (Table 7). Results of one way ANOVA showed significant differences in ALP activity between different days: Day 0, Day7, Day 14 and Day 21. As shown in Bonferonni post hoc analysis revealed that ALP activity of day 21 was significantly higher than day 14, day 7 and day zero, F(3,8) = 649.6, *p* < 0.01. The significant increase in ALP activity as the day of incubation increase proved that the osteogenic potential in day 21 is the best compared to other days and also proved that the hydroxyapatite scaffold is optimal scaffold as it induced the differentiation of cells into osteoblast.

## Discussion

The International Society for Cellular Therapy (ISCT) proposed minimal criteria to define human MSCs based on plastic adherence, differentiation, and the expression of specific antigens. MSCs including BMSC, DPSC and SHED share similar in vitro characteristics including a fibroblast-like morphology, multilineage differentiation and good proliferation capacity. DPSC and SHED were used in this study because they are easier to obtain and less invasive [3]. A study compared DPSC and BMSC in Bio-Oss scaffold reported that both cell types exhibit a similar morphology, proliferative ability, surface marker expression, and trilineage differentiation [21]. However, several studies proved that DPSC and SHED could be survived in vitro for a longer time, exhibited a higher growth rate, and produced an extracellular matrix with less immunosuppressive activity compared to BMSC [22, 23].

The data for identification of DPSC and SHED have been carried out by morphology analysis and stem cell marker. It was observed that the isolated DPSC and SHED at the beginning adhered to the flask and appeared fibroblast-like morphology. After two weeks of culture, the heterogeneous population of cells was observed. Then, they appeared to be homogenous with fibroblast-like morphology toward their following passages which is the main characteristic of MSCs during in vitro culture. A similar finding was demonstrated by Farinawati et al. [24]. Besides, the ISCT proposed positive and negative markers that helped the scientists to distinguish MSCs from other cells. Several studies proved that DPSC and SHED have all the characteristics of MSCs and they positively expressed CD73, CD90, CD105, CD106, CD146, and CD166 markers [13]. However, as a mesenchymal cell, they negatively expressed CD11b, CD19, CD34, CD38 and CD45 [25]. In this study, DPSC and SHED expressed CD73, CD105, CD146, GAPDH and they negatively expressed the hematopiotic markers i.e. CD11b, CD34 and CD45.

The SEM images demonstrated that the granular hydroxyapatite scaffold has a rough surface with unevenly distributed pores with size around 200–500 µm. Wahab et al. [26] reported that the minimum requirement for pore size is approximately 100 µm to assist in migration requirement, cell size and transport. Pore size that are smaller than 100 µm caused hypoxic conditions and induced osteochondral formation before osteogenesis while bigger pore size induced bone tissue formation [26]. Our study is in agreement with the consensus that pore size of more than 200 µm is required for osteoconduction [27]. In addition, the SEM micrographs showed that the surface of the scaffold was covered with elongated, fusiform cells which became denser as they increased to day 21. In complete medium, the cells showed a fibroblastic like morphology compared to the differentiating medium, it appears round like morphology cells which is the morphology of osteoblasts. A similar study observed that the fibroblastic shape of DPSC was turned into a rounded shaped cells (morphology of osteoblast) which was attached to Poly-L-Lactic Acid scaffold (PLLA) [28]. Good cell adhesion of all cells in the compelete and the differentiating medium was confirmed by SEM micrographs. This is similar to a previous study as granular hydroxyapatite scaffold support adhesion and proliferation of pre-osteoblast cells [10]. Furthermore, the extracellular matrix (ECM) became dominant in the SEM micrographs at day 21 and it had covered the scaffold and the pores. The highly developed ECM network appearance is in agreement with the findings of Karadzic et al. [29] who reported that the dominant ECM by day 21 indicates extensive differentiation and good biocompatibility between cells and materials, which is important in tissue engineering.

Alkaline phosphatase (ALP) is an enzyme that's naturally present throughout the body. Levels of this enzyme increase when bones are growing, or whenever bone cells are active. The osteogenic differentiation of mesenchymal stem cells was the first differentiation that was identified by Friedenstein et al. in 1970. They extracted bone marrow stromal cells from the bone marrow and differentiated it into osteoblasts. The osteogenic differentiation using ALP assay occurred in vitro during a period of one month and produced differentiated osteoblast [30, 31]. This study showed that the ALP of the three types of cells peaks on day 21 which indicated that it is the best period for incubation because previous studies such Ammar [32] and Shafiee et al. [33] found also that ALP activity using MSCs was decreased after day 21 when they incubated until 28 day as it is thought to be correlated with maturation of the bone cells as they become Osteocytes. The results also showed that the ALP activity of MC3T3-E1, DPSC and SHED in the differentiating medium was increased by day 7, day 14 and day 21. This is in agreement with the finding of Kuo et al. [34] who reported that the ALP activity of MC3T3-E1 in hemostatic gelatin sponge under osteogenic induction was increased with culture time. Similar studies reported that the ALP of SHED and DPSC was increased as the incubation day increased [5, 29]. It is also proved that the ALP activity of SHED in 2D and 3D culture was higher compared to DPSC. This is in agreement with Wang et al. [25] and Yazid et al. [35] who reported that the ALP activity of SHED was higher than DPSC. In hydroxyapatite scaffold, hydrated calcium phosphate forms a dense apatite layer that mimics natural bone structure. This induces cell differentiation and mineralization in the absence of any osteogenic factor [36]. This study verifies that granular hydroxyapatite scaffold fulfills the criteria of an optimal scaffold for bone tissue engineering as it promoted cell adhesion and induced osteogenesis of MC3T3-E1, DPSC and SHED. This is in agreement with Pereira-Junior et al. [37] who assessed the efficiency of the granular hydroxyapatite scaffold using BMSC and they proved that the gHA scaffold supports the adhesion and the differentiation of BMSC. The limitation of this study is that the osteogenic differentiation was not confirmed by, for example, Alizarin red staining, use other osteogenic specific marker such as Osterix, osteocalcin, BMP, RUNX2 etc., which will be addressed in future study. Furthermore, this study was conducted in vitro, and the findings need to be evaluated also in vivo assays.

## Conclusion

In conclusion, SHED had the highest osteogenic potential as compared to MC3T3-E1 and DPSC. It is the best candidate for tissue engineering as it showed the highest degree of mineralization and the highest abundance of nodules in differentiating medium. Furthermore, as the ALP increased with days, this proved that granular hydroxyapatite scaffold is a suitable scaffold for generating bone-like material because it supports cell adhesion and induces osteogenesis. Future works should be focusing on the rate of resorption using gHA with stem cells in vivo application.

## Data Availability

All data used and analyzed for the current study are available from the corresponding author on reasonable request.

## Abbreviations

DPSC: Dental pulp stem cells
SHED: Stem cells from exfoliated deciduous teeth
gHA: Granular hydroxyapatite scaffold
MgCl_2_: Magnesium chloride
dNTP: Deoxynucleotide4 triphosphates
PBS: Phosphate-buffered saline
Na_2_CO_3_: Sodium carbonate
NaHCO_3_: Sodium bicarbonate
